# The Role of Bioactive Compounds on the Promotion of Neurite Outgrowth 

**DOI:** 10.3390/molecules17066728

**Published:** 2012-06-04

**Authors:** Sandeep Vasant More, Sushruta Koppula, Byung-Wook Kim, Dong-Kug Choi

**Affiliations:** Department of Biotechnology, Research Institute for Biomedical and Health Science, Konkuk University, Chungju 380-701, Korea

**Keywords:** **:** neurite outgrowth, neuroregeneration, neuritogenesis, natural molecules, neurodegeneration

## Abstract

Neurite loss is one of the cardinal features of neuronal injury. Apart from neuroprotection, reorganization of the lost neuronal network in the injured brain is necessary for the restoration of normal physiological functions. Neuritogenic activity of endogenous molecules in the brain such as nerve growth factor is well documented and supported by scientific studies which show innumerable compounds having neurite outgrowth activity from natural sources. Since the damaged brain lacks the reconstructive capacity, more efforts in research are focused on the identification of compounds that promote the reformation of neuronal networks. An abundancy of natural resources along with the corresponding activity profiles have shown promising results in the field of neuroscience. Recently, importance has also been placed on understanding neurite formation by natural products in relation to neuronal injury. Arrays of natural herbal products having plentiful active constituents have been found to enhance neurite outgrowth. They act synergistically with neurotrophic factors to promote neuritogenesis in the diseased brain. Therefore use of natural products for neuroregeneration provides new insights in drug development for treating neuronal injury. In this study, various compounds from natural sources with potential neurite outgrowth activity are reviewed in experimental models.

## Abbreviations

Ntrk1: neurotrophic tyrosine kinase receptor type 1: NGF: Nerve growth Factor,
B-Raf: Serine/threonine-protein kinase: GTPP: Green tea polyphenol
MEKK: mitogen-activated protein kinase kinase kinase: CREB: cAMP response element-binding
MEK: mitogen-activated protein kinase kinase: Rap1: Ras-Proximate-1
ERK1/2: extracellular signal-regulated kinase: Ras: GTPasep21^ras^
p38-MAPK: p38 mitogen-activated protein kinase: ROS: Reactive oxygen species

## 1. Introduction

Neuroregeneration is a concept which encompasses endogenous neuroprotection leading to neuroplasticity and neurite outgrowth [1]. The principal morphological characteristics of neuritogenesis are branching of neurites followed by elongation of axons and dendritic arborization [2]. Neurite genesis and retraction are basically associated with the development and pathogenesis of the nervous system [3]. It has been suggested that reconstruction of the neuronal and synaptic networks in the injured brain is necessary for the recovery of brain functions [4]. It was once believed that nerve regeneration in the mammalian central nervous system (CNS) was irreversible, but recently it has become apparent that damaged neurons do regenerate in an active process under the presence of stimulatory substances such as nerve growth factor (NGF) and brain derived neurotrophic factor (BDNF) [5,6,7,8,9].

Neuritogenic substances hold the promise of therapeutic efficacy in the treatment of neuronal injuries by the virtue of their ability to stimulate outgrowth of neurites from neuronal cells. Because of neurite outgrowth, there is a readjustment in the normal neuronal functions and local circuits in the damaged CNS [10]. Therefore, use of the neurotrophic factors seems to be an important step in the process of neuronal regeneration. Despite of their undeniable positive effects, administration of neurotrophic factors also has some disadvantages like, negligible entry through the blood brain barrier and destabilization by peripheral peptidases [11]. To overcome this hurdle, use of natural products eliciting neuritogenic activity or in combination with neurotrophic substances is currently been focusedas an alternative approach [12]. 

Recent literature suggests that different compounds from natural sources in combination with NGF act synergistically to induce the growth of neurites. Hence natural products may harmonize very well for the treatment of neuronal injury [4,13,14,15,16,17]. Lately, many compounds from natural sources have been demonstrated to possess neurotrophic and neuroprotective abilities (Table 1). Current research also affirms the role of natural products to enhance the neurite outgrowth activity of NGF in various experimental models [10]. In addition to this, other evidences also shows that compounds capable of enhancing the action of neurotrophic factors to stimulate neurite outgrowth may be useful in the treatment of neurological disorders such as Parkinson’s disease (PD) and Alzheimer’s disease (AD) [18,19,20]. Hence, compounds obtained from natural sources can be used for neuroregeneration in various forms of neuronal injury. In the present review, we have focused on the neurite outgrowth activity of several natural compounds or their crude extracts in various forms of neuronal injury.

**Table 1:** Summary of selected compounds from natural sources exhibiting *in vitro and in vivo* neurite outgrowth activity.

Main Biological Source	Compounds	Effective dose	Activity	Ref.
*Panax ginseng*	Ginsenoside Rb1	40 mg	Neuritogenesis in rats	[21]
*Panax ginseng*	Ginsenoside Rg1	10 mM	Survival of dopaminergic neurons	[22]
*Curcuma longa*	Curcumin	10 & 20 μM/ 0.2 mg	Neurite outgrowth in PC12 cells/Neurogenesis in mouse	[23,24]
*Withania somnifera*	Withanoside IV & VI	1 μM	Axon & dendritic extension in rat cortical neurons	[4]
*Camellia sinensis*	EGCG	0.1–1 μM	Neurite outgrowth in PC12 cells	[25]
*Picrorhiza scrophulariiflora*	Picroside I & II	60 μM	Potentiating NGF induced neurite outgrowth in PC12D cells	[26]
Propolis	Artepillin C	10, 20 & 50 μM	Potentiating NGF induced neurite outgrowth	[16]
*Rehmannia glutinosa*	Catalpol	5, 15 & 50 mg	Increase in the number of mouse tyrosine hydroxylase positive cells	[27]
*Citrus depressa*	Nobiletin	100 μM	Neurite outgrowth in PC12 cells	[28]
*Sargassum macrocarpum*	Sargaquinoic acid	1.25–100 ng	Potentiating NGF induced neurite outgrowth in PC12D cells	[29]
*Tripterygium wilfordii*	Tripchlorolide	10^−10^ M	Neurite outgrowth & survival of dopaminergic neurons.	[30]
*Scutellaria baicalensis*	Baicalein	5 μg/ 50 & 200 mg	Neurite outgrowth in PC12cells/Increase & survival of rat TH-positive cells	[31]

## 2. Natural Herbs and their Active Constituents with Neuritogenic Activity

### 2.1. Ginsenosides from *ginseng*

*Panax ginseng* (C. A. Meyer) and *Panax japonicas* (C. A. Meyer) are slow-growing perennial plants with fleshy roots belonging to the *Araliaceae* family. For many years, *P. ginseng* has been used as a medicinal plant in traditional oriental medicine [32]. *P. ginseng* is scientifically reported to have antiulcer [33], DNA damage inhibitory [34], antiapoptotic [35], antiobesity, antiinflammatory, antioxidant [36], antitumor and immunomodulatory [37], antidiabetic [38], hepatoprotective [39], antihypertensive [40], antiamnestic [41] and neuroprotective effects [42]. These bioactivities of ginseng are attributed to the presence of vivid active constituents such as triterpenes, saponins, essential oils (polyacetylenes and sesquiterpenes), polysaccharides, peptidoglycans, nitrogen-containing compounds and various ubiquitous compounds such as fatty acids, carbohydrates, and phenolic compounds [32]. Ginseng has been studied in a number of randomized clinical trials mainly investigating its effects on physical and psychomotor performance, cognitive function, immunomodulation, diabetes mellitus and herpes simplex type II infections [43].

**Figure 1:**
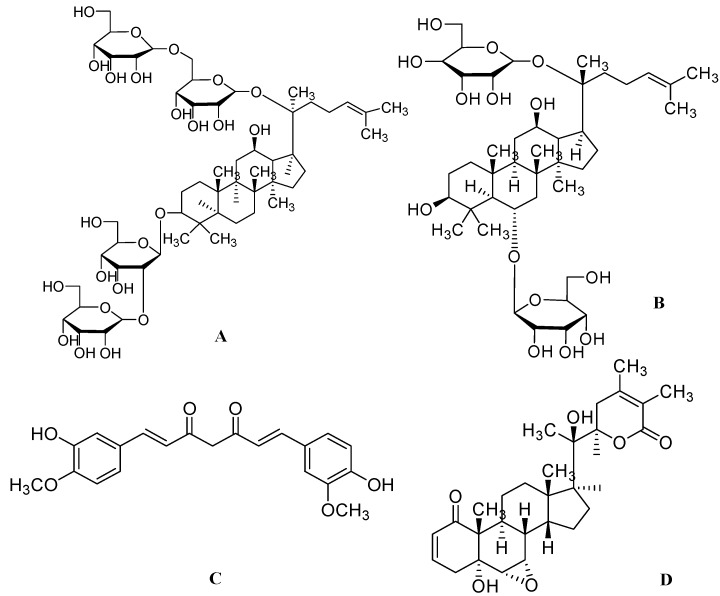
The molecular structures of ginsenoside Rb1 (**A**), ginsenoside Rg1 (**B**), curcumin (**C**) and withanolide A (**D**).

Among 30 major ginsenosides, Rb1 (Figure 1A) and Rg1 (Figure 1B) were found to be the main active ingredients of *Panax* species [44]. Currently, ginseng is well studied for its neurite outgrowth activity in various *in vitro* and *in vivo* models. Recently, Rb1 is reported to induce the expression of BDNF and neurogenesis in rats with cerebral ischemia [21]. Methanolic extracts of ginseng (dried root of *P. ginseng*), red ginseng (steamed and dried root of *P. ginseng*), notoginseng (dried root of *P. notoginseng*) and Ye-Sanchi (dried rhizome and root of *P. vietnamensis* var. fuscidiscus) at a dose of 50 μg/mL increased neurite outgrowth in SK-N-SH cells, with the effects of red ginseng and Ye-Sanchi being particularly significant [4].

Methanolic extract of Ye-sanchi significantly increased neurite outgrowth in human neuroblastoma SK-N-SH cells. Further studies in the isolation yielded 15 neuroactive compounds from this extract. Out of 15 compounds, only four compounds, namely ginsenoside Rb1, ginsenoside Rb3, notoginsenoside Fa and notoginsenoside R4, each at a concentration of 100 μM, were found to produce a significant increase in the percentage of neurite outgrowth in SK-N-SH cells. Moreover, ginsenoside Rb1, ginsenoside Rb3 and notoginsenoside R4 significantly increased the total length of neurites, number of varicosities and sites of synaptic connection. These results suggest that these molecules are certainly useful for promotion of neuritogenesis [45]. Reports also indicated that Rb1 and Rg1 induce neurite outgrowth in cultured rat cerebral cortical neurons [46]. Ginsenoside Rb1 has also been found to potentiate nerve NGF mediated neurite outgrowth of chick dorsal root ganglia [47,48].

Ginsenosides Rb1 and Rg1 at a concentration of 10 mM have not only been found to enhance the survival of dopaminergic neurons by 19% and 14%, but also ameliorated the degenerative changes such as cell swelling and loss of neurites. Both these ginsenosides counteracted the degeneration by 1-methyl-4-phenylpyridine (MPP^+^) and significantly protected lengths and numbers of neurites of tyrosine hydroxylase (TH) positive cells. The stimulatory effects of both ginsenosides on survival of dopaminergic cells may be mediated through improving the energy metabolism and preserving the structural integrity of neurons. Cumulatively, ginsenosides Rb1 and Rg1 are promising molecules for promoting neurite outgrowth activity [22].

### 2.2. Curcumin from *Curcuma longa*

Curcumin from *Curcuma longa L.* of the *Zingiberaceae* family is a commonly used spice with well documented medicinal properties in Indian and Chinese medicine [49]. Curcumin (Figure 1C) has been reported to possess several beneficial bioactivities such as antiinflammatory [50], antioxidant [51], antimutagenic [52], antidiabetic [53], anticancer [54], antiangiogenic [55], antibacterial, antiviral [56], immunomodulatory [57], wound healing [58] and neuroprotective properties [59]. Curcumin is also under investigation for its clinical benefit in AD and colon cancer [60].

Recent reports have shown that extract of crude turmeric protects PC12 cells from an insult of 20 μg/mL of β-amyloid (1–42). Studies on curcuminoids also have reported neurite outgrowth activity at a dose of 10 and 20 μM in PC12 cells. Curcumin could possibly exhibit neurite outgrowth activity by extracellular signal-regulated kinase (ERK) and protein kinase C (PKC) dependent pathways [23]. Furthermore, curcumin at doses of 10 and 20 mg/kg, p.o. have also been shown to increase hippocampal neurogenesis in chronically stressed rats. Curcumin is also reported to significantly prevent stress-induced decrease in BDNF protein levels in the hippocampus [61]. Treatment of curcumin at a dose less than 0.2 mg/kg significantly increases the proliferation of neural stem cells and increases neurogenesis in the hippocampus of C57BL6 mice. Therefore use of curcumin would certainly be beneficial with regard to aging [62] and AD [24] wherein neurogenesis is compromised.

### 2.3. Withanosides from *Withania somnifera*

**Figure 2:**
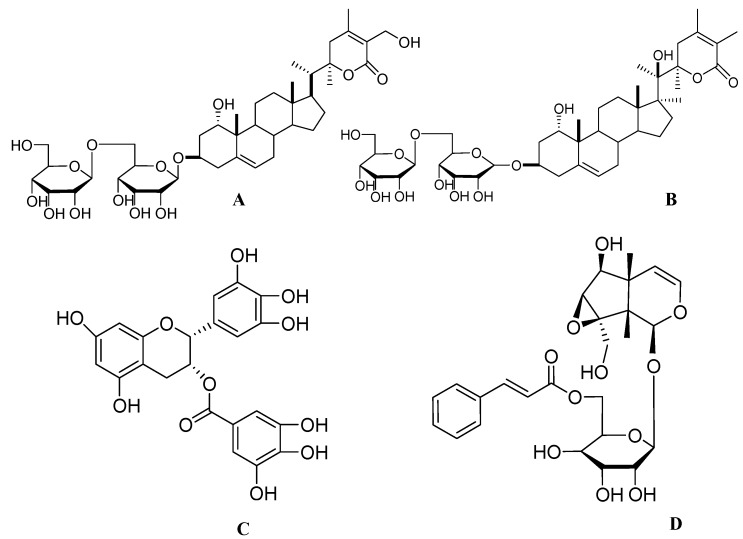
The molecular structures of withanoside IV (**A**), withanoside VI (**B**), epigallocatechin-3-gallate (**C**) and picroside I (**D**).

*Withania somnifera (W. somnifera)*, popularly known as ashwagandha in ayurvedic medicine, belongs to the *Solanaceae* family. This herb has been used traditionally as a tonic and nootropic agent. Beside its traditional uses, ashwagandha also has immunomodulatory [63], antiamnestic [64], antioxidant, antimicrobial [65] and herbicidal activities [66]. *W. somnifera* has been tested in a double-blind placebo-controlled study for rheumatoid arthritis [67]. Majority of the active ingredients are found in the roots of this herb*.* Recent literature has shown that the methanolic extract of ashwagandha at 5 μg/mL induced neurite extension in human neuroblastoma SK-N-SH cells. Further isolation studies revealed some major active constituents such as withanolide A (Figure 1D), withanoside IV (Figure 2A) and withanoside VI (Figure 2B). One μM doses of each compound produced significant neurite outgrowth in dopaminergic SH-SY5Y cells [68]. Treatment of withanoside IV or withanoside VI induced a predominant dendritic outgrowth in cortical neurons, whereas predominant axonal outgrowth was observed after treatment with withanolide A in normal cortical neurons [69]. Withanolide A, withanoside IV and withanoside VI at a concentration of 1 μM prevented both dendritic and axonal atrophy in rat cortical neurons induced by β-amyloid (25–35). Furthermore, treatment with withanosides IV and VI tended to induce the growth of longer dendrites than treatment with withanolide A [4]. Thus ashwagandha provides a new option for promoting neuroregeneration.

### 2.4. Green Tea Polyphenols from *Camellia sinensis*

Green tea obtained from *Camellia sinensis* is one of the oldest beverages, having several beneficial effects against cardiovascular disorders and obesity [70]. Green tea polyphenols (GTPP) are well known for their antioxidant effects. Polyphenols are natural substances present in beverages obtained from plants, fruits and vegetables. Flavonoids form the largest group of polyphenols, which include the flavone, isoflavone, flavanol, flavan, and flavonol subclasses [71]. Among the polyphenols present in GTPP, epigallocatechin-3-gallate (EGCG) (Figure 2C) has been found to possess an array of biological activities such as antioxidant, antibacterial [72], antiinflammatory [73], anticancer [74], antidiabetic [75], antihypertensive [76], and neuroprotective effects [77]. A world leading research group is also planning to start the first clinical trial with EGCG in patients with amyloid light-chain amyloidosis [78].

In a recent report, EGCG alone potentiated NGF induced neurite outgrowth in PC12 cells while other polyphenols present in GTPP, particularly epigallocatechin and epicatechin, did not show neurite outgrowth activity. When epigallocatechin and epicatechin were combined with EGCG, they synergistically promoted neurite outgrowth activity in PC12 cells. On the other hand, GTPP alone did not induce neurite outgrowth to an appreciable extent even at high concentrations of 1–5 µg/mL. However, GTPP in combination with a low concentration of NGF induced neurite outgrowth to an extent equivalent to that achieved with the high concentration of NGF alone. A combination of GTPP and NGF, besides increasing the number of neurites, also increased the branching and length of the neurites. In this case, the neurites grew to a length nearly two to four times longer than the cell body diameter. Another report supporting the above result also showed that EGCG at 0.1–1 μM induced neurite outgrowth in PC12 cells [25]. Cumulatively, these results indicate that GTPP potentiates NGF-induced neuritogenesis and may serve as an optional therapy for neurodegenerative disease [79]. 

### 2.5. Picrosides from *Picrorhiza scrophulariiflora*

**Figure 3:**
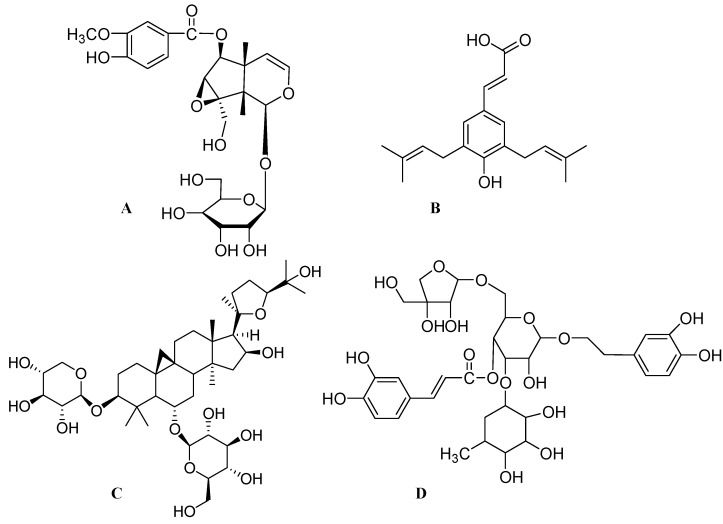
The molecular structures of picroside II (**A**), artepillin C (**B**), astragaloside IV (**C**) and pedicularioside A (**D**).

*Picrorhiza scrophulariiflora* is a perennial traditional herb belonging to the family *Scrophulariaceae*. This herb grows in the high-altitude regions of Tibet, Yunnan and China. The roots of this plant are rich in terpenoids, iridoid glycosides, phenolic glycosides and phenylethanoid glycosides [80]. The dried rhizomes of *Picrorhiza* have long been used in Southeast Asia to treat inflammatory diseases such as arthritis and asthma [81]. Picrorhiza is scientifically reported to have immunomodulatory [82], hepatoprotective [83], antioxidant, antineoplastic [84], choleretic activity [85], hypolipidemic [86], antineuropathic [87] and neuroprotective activities [88]. Crude powder of *Picrorhiza* has also shown significant effect in a randomized, double-blind placebo controlled trial in patients diagnosed to have acute viral hepatitis [83]. Recently, picrosides I (Figure 2D) and II (Figure 3A) were shown to possess neuritogenic activity. Treatment of PC12D cells with picroside I and II at 60 μM did not show any neurite elongation. But combination of picrosides at 60 μM combined with NGF at 2 ng/mL produced a significant neurite outgrowth in PC12D cells [26]. Other studies have also reported that, picrosides I and II showed a concentration-dependent (more than 0.1 μM) enhancement of basic fibroblast growth factor (bFGF) at 2 ng/mL, staurosporine at 10 nM and dibutyryl cyclic AMP (dbcAMP) at 0.3 mM—induced neurite outgrowth from PC12D cells. Further mechanistic studies found that this effect was due to amplification of the intracellular Mitogen-activated protein kinase (MAPK)-dependent signaling pathway [10].

## 3. Other Miscellaneous Compounds and Extracts from Natural Sources that Show Neuritogenic Activity

Propolis is a resinous natural product obtained from various botanical sources [89]. Propolis has been shown to have a wide range of biological activities like antioxidant and anti-ischemic activity [90,91,92], principally due to the presence of flavonoids and caffeic acid phenethyl ester (CAPE) [93]. Recently a phase II clinical trial has shown effectiveness of a mouthwash containing propolis for the control of plaque and gingivitis [94]. The active ingredient in propolis is a low-molecular-weight polyphenolic compound named artepillin C (Figure 3B) (3,5-diprenyl-4-hydroxycinnamic acid). Different studies have also reported artepillin C to possess antibacterial activity and antitumor activity *in vitro* [95,96].

Neurite outgrowth activity of artepillin C was evaluated in PC12 mutant cell line (PC12m3) in which NGF-induced neurite outgrowth is impaired. The degree of neurite outgrowth shown by NGF alone in PC12m3 cells is insignificant. But artepillin C in combination with NGF significantly enhanced the neurite outgrowth in PC12m3 cells. The degree of neurite outgrowth induced by artepillin C at concentrations of 10, 20 and 50 µM were approximately 5.4, 7.4 and 6.0 times greater than that induced by NGF alone. Further studies revealed that artepillin C induced neurite outgrowth in PC12m3 cells was mediated by the p38-MAPK signaling pathway [16].

Astragaloside IV (AS-IV) (Figure 3C) is the active ingredient extracted from the dried root of *Astragalus membranaceus (A. membranaceus)* of the *Fabaceae* family. *A. membranaceus* is well-known in Traditional Chinese Medicine (TCM) and is used as a key herbal remedy for its antiinflammatory, antioxidative, cardio protective and immune system stimulating activities [97]. A very recent double-blind, placebo-controlled, randomized study has reported *A. membranaceus* to enhance recovery from hemorrhagic stroke [98]. The active ingredient AS-IV, extracted from the dried root of *A. membranaceus* was reported to inhibit free radicals, decrease lipid peroxidation and increase anti-oxidant enzymes [99]. Treatment of primary nigral cells (PNCs) with 6-hydroxyl dopamine (6-OHDA) along with AS-IV at a concentration of 50 and 100 µM showed most of the dopaminergic neurons were intact and sprouting. AS-IV also rescued primary dopamine neurons from 6-OHDA induced neurotoxicity and degeneration. Treatment of AS-IV alone at a dose of 100 and 200 µM also induced extensive neurite outgrowth from TH neurons. AS-IV also enhanced axonal regeneration and reconstruction of neuronal synapses and prevented β-amyloid (25–35) induced neuronal death [100]. Further mechanistic study found that regenerative and neuroprotective effect of AS-IV was due to its antioxidant activity [101].

*Buddleia lindleyana (B. lindleyana)* is a well-known Chinese medicinal herb belonging to the *Scrophulariaceae *family*. B. lindleyana* is widely used as an antiinflammatory medicine for the treatment of swelling, lung pain and wounds in Southwest China [102]. Pedicularioside A is the main active ingredient in *B. lindleyana.* Chemically, pedicularioside A (Figure 3D) is a phenylethanoid glycoside. Treatment of pedicularioside A offered a significant protective effect to cultured mesencephalic neurons exposed to MPP^+^. Pedicularioside A at a concentration of 16 μM produced increased number of viable cells as compared to control which suggests that pedicularioside A might have a neuroprotective effect. Pretreatment of pedicularioside A before MPP^+^ resulted in cells that displayed quite elongated and arborized processes, with the number of TH positive neurons decidedly higher than those seen in MPP^+^ control cultures. Moreover, the TH positive neurons in the cultures pre-treated with pedicularioside A were seen to have longer and more developed processes. These results indicate that pedicularioside A via its neuritogenic effect, inhibits MPP^+^ induced death and loss of dopaminergic neurons [103].

**Figure 4:**
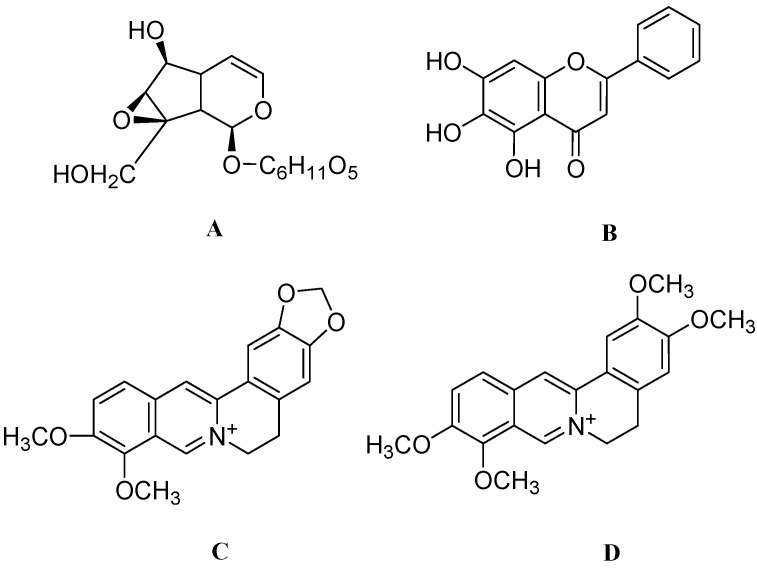
The molecular structures of catalpol (**A**), baicalein (**B**), berberine (**C**) and palmatine (**D**).

Catalpol (Figure 4A) is basically an iridoid extracted and purified from the *Rehmannia glutinosa* belonging to the *Scrophulariaceae* family. Some preliminary data in animal models suggests its antiamnestic effect [104] and amelioration of β-amyloid induced degeneration of cholinergic neurons by elevating BDNF levels [105]. In a chronic 1-methyl-4-phenyl-1,2,3,6-tetrahydropyridine (MPTP) mouse model it was found that, catalpol at doses of 5, 15 and 50 mg/kg/day significantly raised the average number of TH positive neurons in substantia nigra pars compacta (SNpc) and striatal dopamine transporter (DAT) density. Catalpol significantly increased glial derived neurotrophic factor (GDNF) protein and GDNF mRNA levels as compared to control. Since GDNF was mainly synthesized in astrocytes, the role played by astrocytes in the neuroregenerative effect of catalpol is important. Taken together, it is evident that catalpol mitigates the neurodegenerative decline of nigral TH neurons and promotes neurite outgrowth via elevating GDNF levels [27].

*Scutellaria baicalensis* is a well known Chinese medicinal herb belonging to the *Lamiaceae* family*.* Baicalein (Figure 4B) is the major active ingredient found in the roots of this herb*.* Baicalein is reported to be a potent antiinflammatory and antioxidant and has also been regarded as a 12/15-lipoxygenase and xanthine oxidase inhibitor [106,107]. Pretreatment of 6-OHDA lesioned PC12 cells with baicalein at concentrations of 0.5 and 5 μg/mL helps to form polygonal shapes in PC12 cells. This effect is very similar to the effect that is shown with NGF alone. Moreover, treatment of baicalein (50 and 200 mg) in an animal model of PD significantly increases TH positive neurons in SNpc. These results suggested that baicalein rescues dopaminergic neurons in 6-OHDA lesioned animals due to its neurite outgrowth activity [31].

**Figure 5:**
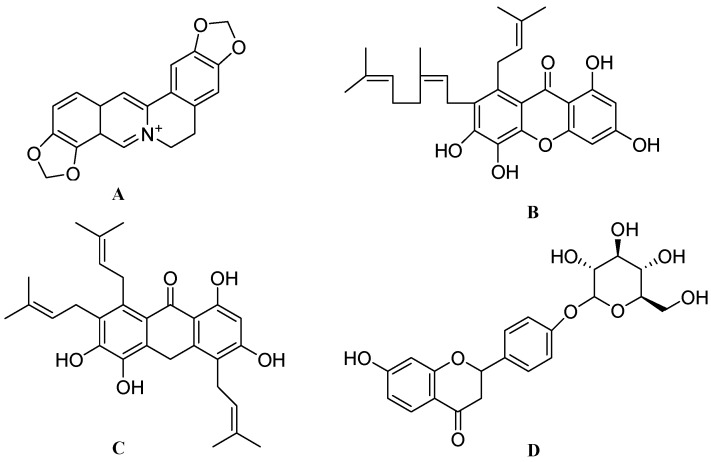
The molecular structures of coptisine (**A**), garciniaxanthone-E (**B**), 1,3,5,6-tetrahydroxy-4,7,8-tri(3-methyl-2-butenyl) xanthone (**C**) and liquiritin (**D**).

*Coptidis rhizoma* is a Chinese perennial herb belonging to the *Ranunculaceae* family. This herb has an array of antimicrobial, antifungicidal and antimalarial activities [108,109]. The active ingredients are alkaloidic in nature and are mainly present in the rhizome of this herb. Recently a randomized controlled trial has reported the safety and effectiveness of JinQi-Jiangtang tablets containing alkaloids from *Coptidis rhizome* for pre-diabetes [110]. Treatment with a methanolic extract (50 and 100 μg/mL) of *Coptidis Rhizoma* effectively enhanced neurite outgrowth in PC12 cells. This extract yielded 3 active compounds after isolation namely, berberine (Figure 4C), palmatine (Figure 4D) and coptisine (Figure 5A). It is found that treatment to PC12 cells with berberine at 5 and 25 μg/mL enhanced the NGF-induced neural differentiation in PC12 cells. Palmatine and coptisine at 5 and 25 μg/mL had a weaker NGF-enhancing effect on PC12 cells. The mechanism of differentiation still remains to be elucidated but certainly these alkaloids may become promising agents in neuroregeneration [111].

*Garcinia xanthochymus* (*G. xanthochymus*) is a medicinal plant belonging to the family *Guttiferae*. *G. xanthochymus* is found to be rich in prenylated xanthones. The Garcinia species are rich in prenylated xanthones. One of the notable lead molecules among those is gambogic acid, which has been studied in phase II clinical trials in China as a prominent anticancer drug candidate [112]. Xanthone constituents have been reported to possess several biological activities such as antibacterial [113], antimalarial [114] and inhibition of cyclooxygenase and prostaglandin E2 [115]. Methanolic extract of *G. xanthochymus* contains two neuroactive compounds, garciniaxanthone-E (Figure 5B) and 1,3,5,6-tetrahydroxy-4,7,8-tri(3-methyl-2-butenyl)xanthone (Figure 5C). Treatment of the latter compound at 1–3 μM and garciniaxanthone-E at 1–10 μM did not promote neurite outgrowth from PC12D cells in the absence of NGF, but treatment of PC12D cells with 1,3,5,6-tetrahydroxy-4,7,8-tri(3-methyl-2-butenyl)xanthone at 3 μM and garciniaxanthone-E at 10 μM significantly increased the NGF-induced (2 ng/mL) proportion of neurite-bearing cells by 8.3% and 24.9% respectively. These results show the neuritogenic potential of garciniaxanthone-E and 1,3,5,6-tetrahydroxy-4,7,8-tri(3-methyl-2-butenyl) xanthone in combination with NGF [116].

*Glycyrrhizae radix (G. radix)*, belonging to the family *Leguminosae* is a frequently used herb for detoxification purposes and to treat injury or swelling in traditional oriental medicine. Liquiritin (Figure 5D) was found to be main active ingredient in *G. radix,* having a range of pharmacological effects including neuroprotection [117,118]. Liquiritin used as a cream is reported to be effective in one of the clinical trials for melasma [119]. Scientific reports have shown that *per se* treatment of liquiritin did not trigger any differentiation of PC12 cells. Liquiritin (5–100 mg/mL) in the presence of a low dose NGF (2 ng/mL) produced a dose-dependent increase in neurite outgrowth in PC12 cells [117].

**Figure 6:**
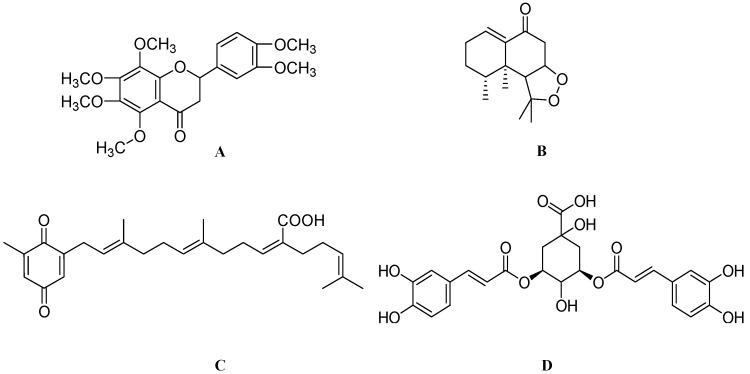
The molecular structures of nobiletin (**A**), nardosinone (**B**), sargaquinoic acid (**C**) and (−)-3,5-dicaffeoyl-muco-quinic acid (**D**).

Nobiletin (Figure 6A) is a low-molecular-weight flavonoid from *Citrus depressa* and belongs to the family *Rutaceae*. Nobiletin has been shown to possess antiatherogenic effect [120]. Treatment of nobiletin (100 μM) induced neurite outgrowth in PC12D cells. Neurite outgrowth shown by nobiletin is due to its persistent increase in phosphorylation of mitogen-activated protein kinase kinase (MEK) leading to a sustained rise in phosphorylation of ERK1/2. Furthermore, phosphorylation of ERK1/2 was accompanied by a transient augmentation of cAMP response element-binding (CREB) phosphorylation and CRE dependent transcription. Nobiletin also caused a preferential inhibition of Ca^2+^/Calmodulin (CaM)-dependent phophodiesterase-1 (PDE1) activity. Inhibition of PDE1 thereby increases intracellular 3'-5'-cyclic adenosine monophosphate (cAMP) concentration to activate Protein Kinase A (PKA). These results certainly provide us with a new molecule having potential neuritogenic activity [28]. 

The roots and rhizomes of *Nardostachys chinensis* (*N. chinensis*), belonging to the family *Valerianaceae*, have long been used for stomach complaints and sedative purposes in oriental medicine [121]. Nardosinone (Figure 6B) is the main active ingredient obtained from the dried roots of *N. chinensis*. Individual treatment of nardosinone at high concentrations of 100 μM did not induce neurite outgrowth from PC12D cells while, *per se* treatment of NGF at 2 ng/mL induced little neurite outgrowth from PC12D cells. Combination of nardosinone with NGF at 2 ng/mL induced neurite outgrowth in PC12D cells in a concentration dependent manner. PC12D cells exposed to nardosinone at 100 μM with NGF at 2 ng/mL formed long neurites which extended to the neighboring cells over distances of 50 μm. Therefore nardosinone may serve as a useful compound for its neuritogenic potential [122].

Sargaquinoic acid (MC14) (Figure 6C) is a low molecular weight plastoquinone isolated from the marine brown alga *Sargassum macrocarpum* [29,123]. Treatment of PC12D cells with MC14 at 3 μg/mL in the presence of NGF at 1.25–100 ng/mL showed a dose-dependent enhancement in the proportion of neurite-bearing cells. Even the number of neurite branching and average neurite length in the MC14 treated PC12D cells was comparable to those induced by a higher dose of NGF (50 ng/mL). Further studies revealed PKC and MAPK-mediated signaling pathways are involved in the NGF dependent neurite outgrowth promoting activity of MC14 in PC12D cells [29]. These findings clearly demonstrate that MC14 can biochemically and morphologically promote the NGF-induced differentiation of PC12D cells [124].

*Aster scaber* T (*A. scaber)* belonging to the family of *Asteraceae*, has long been used in traditional Korean and Chinese medicine to treat bruises, snakebites, headache and dizziness [125,126]. The main active ingredient isolatedfrom *A. scaber*, which induces neurite outgrowth in PC12 cells is (−)-3,5-dicaffeoyl-muco-quinic acid (DQ, Figure 6D). Treatment with DQ at 1, 5 and 10 μM/mL to PC12 cells demonstrated neurite outgrowth and neuronal differentiation in a dose-dependent manner. The potency of DQ at 10 μM was similar to that of NGF at 50 ng/mL. Further studies found that DQ causes the activation of ERK1/2 and phosphatidylinositol 3-kinase (PI3K) in the neurotrophic tyrosine kinase receptor type 1 (Ntrk1) dependent pathway which further leads to the differentiation of PC12 cells [11].

**Figure 7:**
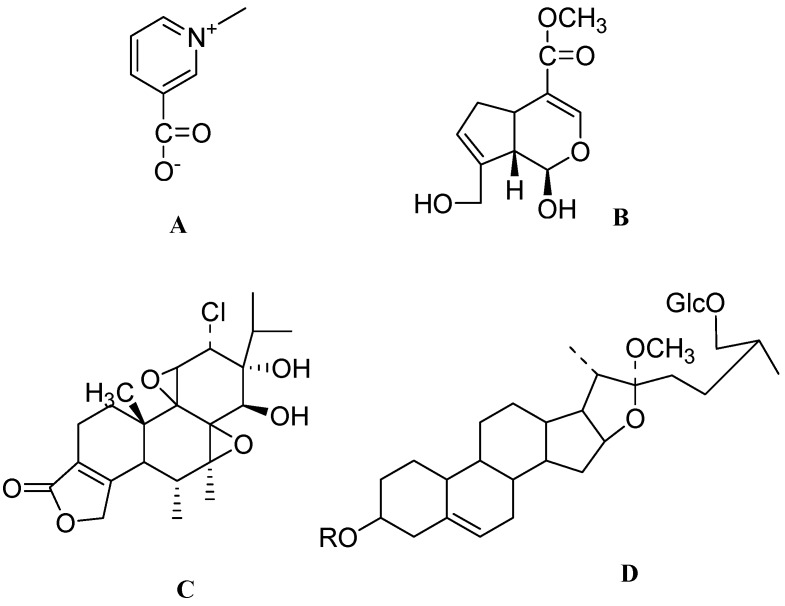
The molecular structures of trigonelline (**A**), genipin (**B**), tripchlorolide (**C**) and R=α-L-rha*p* (1→4)-α-L-rha*p* (1→4) [α-L-rha*p* (1→2)]-β-D-glc*p* (**D**).

Trigonelline (Figure 7A) is an alkaloid found in beans of *Coffea arabica* and belongs to the family of *Rubiaceae.* Coffee is generally consumed as a stimulant drink which not only acts on the CNS but also has an effect on cardiovascular function [4]. Recently in a randomized crossover trial in healthy men, ingestion of trigonelline led to decrease in glucose and insulin concentrations after an oral glucose load [127]. 

Treatment with methanolic extract of coffee beans significantly increases the formation of neurites in human neuroblastoma SK-N-SH cells. Trigonelline was identified as the active ingredient of the extract and *per se* treatment of trigonelline at 30 μM increases the percentage of cells with neurites in SK-N-SH cells. In addition to this, trigonelline also showed a dose-dependent (30 and 100 μM) decrease in dendritic and axonal atrophy induced by β-amyloid in rat cortical neurons [4,128].

Genipin (Figure 7B) is the main active ingredient obtained from an extract of *Gardenia fructus*. Genipin is basically an iridoid and chemically an aglycone of geniposide. Iridoid compounds have been reported to possess various biological activities such as antimicrobial [129], anticancer [130], hemodynamic [131], choleretic [132] and hepatoprotective activity [133]. Treatment of genipin at 5 μg/mL on Neuro2a cells significantly induces neurite outgrowth through a nitric oxide (NO)-cyclic guanosine monophosphate (cGMP)-protein kinase G (PKG) signaling pathway which is then followed by ERK1/2 activation in Neuro2a cells. These findings suggest that genipin is a novel compound having a promising neuritogenic effect [134].

*Tripterygium wilfordii* Hook F (*T. wilfordii*) is a Chinese traditional herb belonging to the family *Celastraceae*. In addition of being used in the treatment of rheumatoid arthritis, this herb is also known for its antiinflammatory and immunosuppressive properties [135]. In a 20-week placebo-controlled study of ethanolic extract of Tripterygium wilfordi hook F (TWHF), a dose-dependent effect was seen in patients with rheumatoid arthritis [136]. One of the active ingredients of *T. wilfordiiis* is tripchlorolide (TW397) (Figure 7C). Chemically TW397 is a diterpene triepoxide and structurally analogous to the major active ingredient triptolide. Treatment of mesencephalic neurons with TW397 at 10^−10^ M promoted neurite elongation by 43% as compared to a vehicle treated group and also increased expression of BDNF mRNA. TW397 at nanomolar concentrations exhibited potent neurotrophic effects and also protected dopaminergic neurons from degeneration induced by both MPP^+^ and 6-OHDA model [30].

*Smilax bockii* Warb belonging to the family of *Liliaceae* is used to treat rheumatoid arthritis and some women-related ailments in TCM [137]. Scientific studies of this plant also showed its antiinflammatory activity [138]. The active ingredient in this herb is chemically a steroid saponin with an R group as α-L-rha*p* (1→4)-α-L-rha*p* (1→4) [α-L-rha*p* (1→2)]-β-D-glc*p* (Figure 7D). Although treatment with this compound alone at 6, 20 and 60 μg/mL did not show any effect on neurite outgrowth in PC12D cells, combination of 60 μg/mL with NGF (2 ng/mL) showed a significant increase in the proportion of neurite-bearing cells in PC12D cells [138].

**Figure 8:**
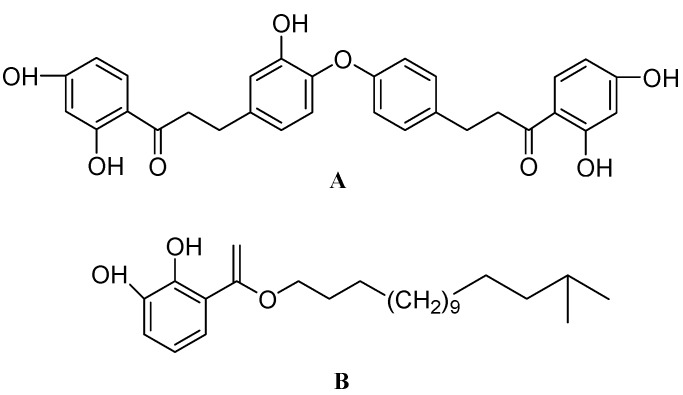
The molecular structures of littorachalcone (**A**) and gentiside C (**B**).

*Verbena littoralis*, popularly known as seashore vervain or Brazilian vervain, belongs to the *Verbenaceae* family. Currently *Verbena littoralis* is used in traditional Central and South American folk medicine against fever, gastrointestinal disorders and some sexually transmitted diseases [139]. The active ingredient in *Verbena litoralis* was found to be littorachalcone (Figure 8A). Littorachalcone is chemically a dimeric dihydrochalcone. It was observed that *per se* treatment of littorachalcone (1–30 μM) to PC12D cells did not affect their morphology, but reasonably enhanced the NGF (2 ng/mL) induced increase in the proportion of neurite bearing cells [140].

*Gentiana rigescens* Franch (*G. rigescens*) belonging to the family *Gentianaceae*, is a well known Chinese medicinal herb. Gentisides found in *G. rigescens* were found to induce neurite outgrowth in PC12 cells. Out of 11 different gentisides, only gentiside A, gentiside B and gentiside C (Figure 8B) exhibited neurite elongation in PC12 cells. Gentiside C (0.03–1 M) showed a dose dependent increase in neurite elongation. Treatment with gentiside C at a concentration of 1 M to PC12 cells also produced long multipolar and bipolar neuritic outgrowths which were similar to those produced following treatment with NGF at 40 ng/mL [141,142].

*Silybum marianum* (milk thistle) is a member of the aster family (*Asteraceae or Compositae*) which includes daisies and thistles such as the common thistle and artichoke. Milk thistle is an herbal supplement that is claimed to have anticancer activity [143] and also beneficial in treating liver [144] and immune disorders [145]. Derivatives of milk thistle have been used over 2,000 years for their beneficial effects. Milk thistle contains many different chemicals, among which silymarin has been found to abate oxidative stress and apoptosis [146]. Silymarin is also been reported to show good results in a double-blind controlled study for its effect on alcoholic liver disease [147]. Milk thistle extract at 100 μg/mL significantly increased the rate of neurite outgrowth in PC12 cells. This neurotrophic effect suggests a potential for the use of milk thistle extract as a neuroprotective or neurotrophic agent in the treatment of neurological disorders [148].

*Ganoderma lucidum* also known as ganoderma or lingzhi belongs to the family *Ganodermataceae*. Ganoderma is a medicinal Chinese mushroom which possesses tumoricidal [149,150] and immunomodulatory activities [151,152]. Treatment of PC12 cells with ganoderma extract at 50 and 100 mg/mL produced neuronal phenotypes including compaction of cell bodies and the extension of neurites. More importantly, ganoderma extract also protected neurons from apoptosis induced by NGF (50 ng/mL) withdrawal. This activity was found to be due to activated MAPK and CREB pathways that are important in regulating the growth and differentiation of PC12 cells [153].

*Melia toosendan* is a well-known TCM effective for the treatment of malaria and stomach aches caused by roundworm [154]. Treatment of PC12 cells with the crude fruit extract of *M. toosendan* at 30 μg/mL resulted in more than 90% of the PC12 cells bearing numerous and elongated neurite extensions. This effect of *M. toosendan* is mediated by the activation of PKC and ERK1/2 signaling pathway. Taken together, these findings show the presence of neuroactive compounds in *M*. *toosendan* that promote neuronal differentiation [155].

Extract of *Cuscuta chinensis* (*C. chinensis*) is one of the important TCM used as a liver tonic. The herb has various biological effects like antiaging and cognition enhancement [156]. Treatment of PC12 cells with *C. chinensis* glycoside (200 mg/L) increased the proportion of neurite-bearing cells and also transformed them to neuronal cells. Further mechanistic studies revealed that MAPK-mediated signaling pathways are involved in neuronal differentiation [157]. 

In summary, most of the compounds evaluated for their neuritogenic activities are derived from plant sources. Among all the majority of *in vitro* models mentioned, neurite outgrowth was basically induced by compounds from natural sources alone or in combination with neurotrophic factors such as NGF. It is also evident from the reports that these compounds show a synergistic effect in their neuritogenic action when combined with NGF.

**Figure 9:**
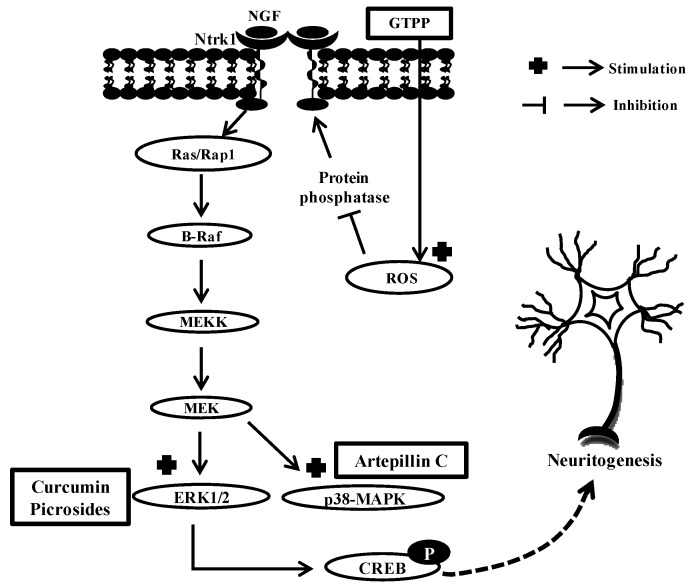
Schematic represents the main molecular mechanisms involved in neurite outgrowth shown by molecules from natural origin.

Based on several reports, compounds from natural sources basically act intracellularly by causing amplification in some specific downstream MAPK signaling pathways like ERK1/2 and p38-MAPK, leading to phosphorylation of CREB and resulting in neurite formation (Figure 9). Another mechanism involved in the induction of neuritogenesis by compounds from natural sources is by generating sublethal levels of reactive oxygen species (ROS) inside the cell. ROS inactivates phosphatase and increases the phosphorylation of Ntrk1 and subsequent activation of ERK1/2 to increase the expression of neuronal marker genes and potentiates NGF-induced neuritogenesis (Figure 9) [79]. 

Natural compounds have also been found to increase levels of neurotrophic factors like GDNF and BDNF [27,30,61]. Increase in GDNF and BDNF levels further adds to neurite outgrowth activity of natural compounds. Despite of the significant increase in our knowledge of the signaling events activated by various compounds from natural sources, many other mechanisms can still be addressed for a comprehensive understanding of neurite outgrowth activity.

## 4. Conclusions

Herbal therapies are one of the most commonly used forms of complementary and alternative medical (CAM) therapies [158]. Discovery of neurogenesis in the adult brain has given attractive therapeutic perspectives for a variety of brain injuries. Recently, a large number of natural products and their isolated compounds possessing neuritogenic activity have been discovered. However, the total acceptance of plant-derived drugs and phytotherapy in medicine can only occur if these products fulfill the same criteria of efficacy, safety and quality control as synthetic products [159]. As compared to synthetic molecules, natural products are structurally diverse and complex with more stereogenic centers and fewer halogen or nitrogen atoms. Naturally active substances usually are good lead molecules, but unlikely to meet the demands for druggability. Therefore, it is necessary to modify and optimize these structural phenotypes. The strategy of structural modification will help us to increase potency, selectivity, improve physico-chemical, biochemical and pharmacokinetic properties and eliminate or reduce side effects [160].

In spite of all the difficulties and limitations, the reviewed current experimental data certainly provides some hope in the identification and understanding of natural products and their active compounds that might be able to induce neurite outgrowth in humans. A move over from *in vitro* cell line studies to *in vivo* animal experiments and eventually human trials is essential. Contribution by the compounds from natural sources to presently available drug is really significant. Some of drugs obtained from natural origin are morphine, codeine, digoxin, quinine, vincristrine and atropine [161,162]. 

Though the available information is discrete, we believe that developments in the near future will allow a better understanding of the molecular mechanisms underlying the complex interplay between neuritogenic compounds from natural sources and various factors regulating neurite outgrowth, thereby making it possible to develop drugs promoting neuronal regeneration. Each piece of knowledge gained from these investigations will help in understanding neuritogenesis, a central process in the human brain, which presents itself as one of the most fascinating and complex subjects in neuroregeneration.
